# One Plus One Makes Three: Triangular Coupling of Correlated Amino Acid Mutations

**DOI:** 10.1021/acs.jpclett.1c00380

**Published:** 2021-03-24

**Authors:** Martin Werner, Vytautas Gapsys, Bert L. de Groot

**Affiliations:** † Computational Biomolecular Dynamics Group, Max-Planck Institute for Biophysical Chemistry, Am Fassberg 11, 37077 Göttingen, Germany

## Abstract

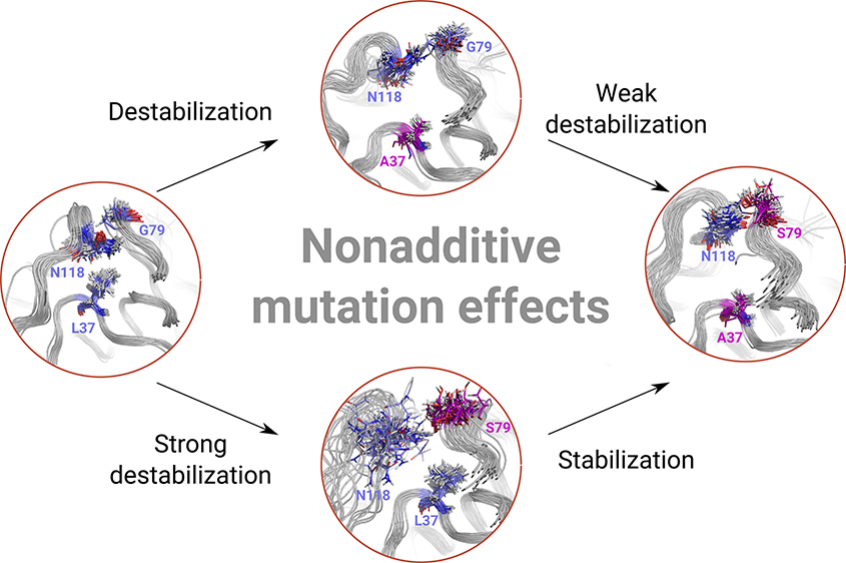

Correlated mutations have played a pivotal role in the recent success in protein fold prediction. Understanding nonadditive effects of mutations is crucial for altering protein structure, as mutations of multiple residues may change protein stability or binding affinity in a manner unforeseen by the investigation of single mutants. While the couplings between amino acids can be inferred from homologous protein sequences, the physical mechanisms underlying these correlations remain elusive. In this work we demonstrate that calculations based on the first-principles of statistical mechanics are capable of capturing the effects of nonadditivities in protein mutations. The identified thermodynamic couplings cover the short-range as well as previously unknown long-range correlations. We further explore a set of mutations in staphyloccocal nuclease to unravel an intricate interaction pathway underlying the correlations between amino acid mutations.

The existence of correlated mutations is well established and has been investigated at the sequence level.^1^ Recently, a groundbreaking achievement was made in the protein folding prediction challenge, where Google DeepMind’s AlphaFold (superseded by AlphaFold2) system outperformed other approaches using a machine learning algorithm exploiting the knowledge of the correlated mutations.^2,3^ While the machine learning algorithm was able to infer relevant inter-residue contacts, the underlying physical mechanisms for these couplings remain elusive.

To learn about the physical nature of the correlations between amino acids, it is convenient to explore the effects of a perturbation by mutation. The introduction of a mutation can alter certain properties of a protein such as its thermostability or binding affinity. The change of the corresponding free energy upon mutation is defined as *ΔΔG*_WT_^A^ (Figure 1a) with respect to the property of interest in the mutational state A and the wild type, respectively. In the case of a double mutation the resulting effect can be significantly different from the sum of the single mutation effects. A measure for this deviation is the nonadditivity δ_WT_^AB^ of the corresponding free energies (Figure 1b).

**Figure 1:**
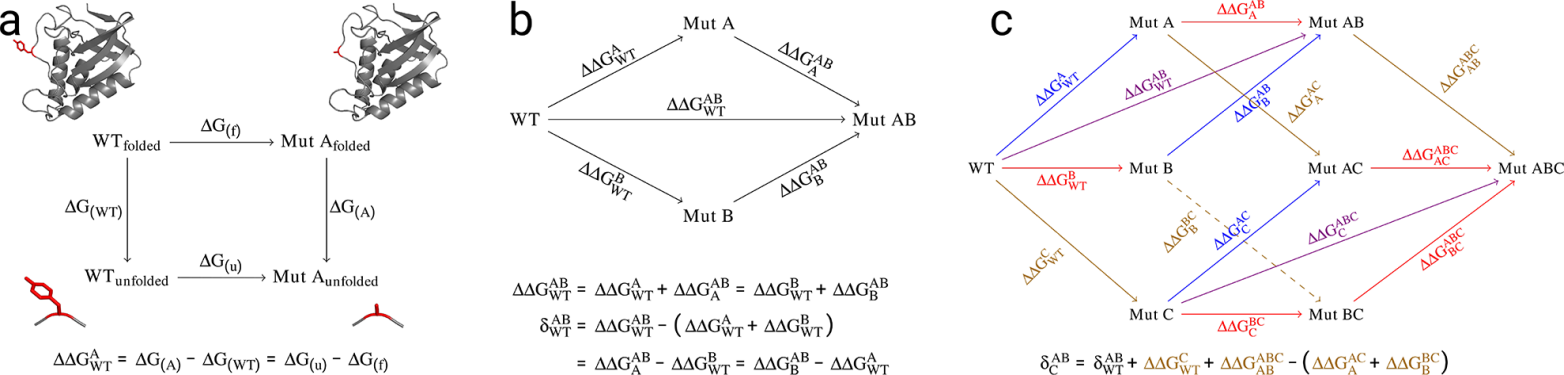
Thermodynamic cycles. (a) Alchemical free energy calculation cycle for the calculation of the ΔΔ*G* of unfolding upon amino acid mutation. Δ*G*_(A)_ and Δ*G*_(WT)_ denote the free energy changes. (b) Double mutant cycle with three different pathways to calculate a double mutation free energy and the corresponding nonadditivity. The subscripts in the equation denote the reference state, while superscripts show the mutational target state. (c) Triple mutant box containing the effect of an external third mutation C on the double mutant cycle of the mutations A and B. Mutation A is highlighted in blue, mutation B in red, mutation C in gold, and the combined mutation of A and B in violet. δ_C_^AB^ can be calculated using the equation in (b) by replacing the reference state from WT to C. Alternatively, (c) shows how δ_C_^AB^ can be expressed by using contributions from the WT protein nonadditivity (δ_WT_^AB^) and single mutations of different protein reference states only, namely, introducing mutation C into the WT protein, as well as its mutated variants A, B, and AB (derivation in the Supporting Information (SI), Figure S1).

In the case of δ_WT_^AB^ = 0 the mutations are perfectly additive. If δ_WT_^AB^ ≠ 0 the two mutations are referred to as being correlated and exhibit a thermodynamic coupling. Such a situation is expected for residues in close spatial contact as the resulting effect can be strongly influenced by the direct interactions between the amino acid side chains involved in the mutations. For distant mutational pairs, the additivity of free energies is widely assumed as it is commonly exploited in the construction of protein contact maps.^4,5^

However, several studies showed that some thermodynamic couplings persist over large distances in space, which can not be explained by direct interactions of the corresponding amino acids.^6−9^ The long-range mutation effects in proteins are of particular interest as they may have significant contributions to enzymatic activity,^10,11^ protein folding,^12^ or ligand binding affinity.^13^ In coevolution analyses coupled mutations can be traced down by multiple sequence alignments. As such, particular focus has been put on the evolutionary development pathways of correlated amino acid mutations and their conservation among protein families.^14−16^ While explanations for this phenomenon reached from mediated interaction between the involved residues^17,18^ over the concept of “spheres of perturbation”^6,19,20^ up to the idea of coupled rigid clusters,^9^ none of these could yield a conclusive understanding of long-range nonadditivity.

In the current work we develop a rigorous approach to quantify nonadditivities in protein mutations using staphyloccocal nuclease as a model system. We further demonstrate that the rigorous physical model is able to recover the nonadditive effects: neither empirical scoring nor machine learning that is not explicitly trained to reproduce this property was able to yield a reliable prediction for a large set of staphyloccocal nuclease nonadditivities. Subsequently, our calculations allowed unveiling the physics behind the correlated interactions between amino acids and predict the effects of their mutation.

Staphyloccocal nuclease (SNase) presents a convenient model system to investigate nonadditive mutations, as in an extensive screen Green and Shortle have identified a large number of nonadditive amino acid mutations experimentally.^6^ We have used an alchemical amino acid mutation protocol^21,22^ based on atomistic molecular dynamics simulations to recapitulate the experimentally measured changes. The alchemical approach presents a rigorous method to compute free energy differences relying on the first-principles of statistical mechanics and allowing circumvention of computationally expensive sampling of the protein folding process (for more details see the SI and ref (23)). In total we probed 18 single and 52 double amino acid mutations. In addition, we validated the method on other proteins, namely, a less well-defined set of barnase and a small set of available myoglobin nonadditivities (see SI).

The thermodynamic cycles in Figure 1a and Figure 1b describe computation of stability changes and nonadditivities, respectively. The more complex cycles considered in Figure 1c allow calculation of the nonadditivity modifications by the third mutation. In addition to the alchemical calculations we also probed predictions based on the empirical energy function of FoldX^24^ and the machine learning approach of the MAESTROweb Web server,^25^ as representatives from a large variety of protein stability predictors (see review^26^). Detailed description of the computational methods and simulation parameters are provided in the SI.

For the single mutations of staphyloccocal nuclease (Figure 2, left column), all the approaches reach state-of-the-art accuracy in comparison to the experimental measurement. The mean absolute difference between calculation and experiment (also termed as average unsigned error, AUE) is lower than 4.184 kJ/mol (1 kcal/mol).

**Figure 2:**
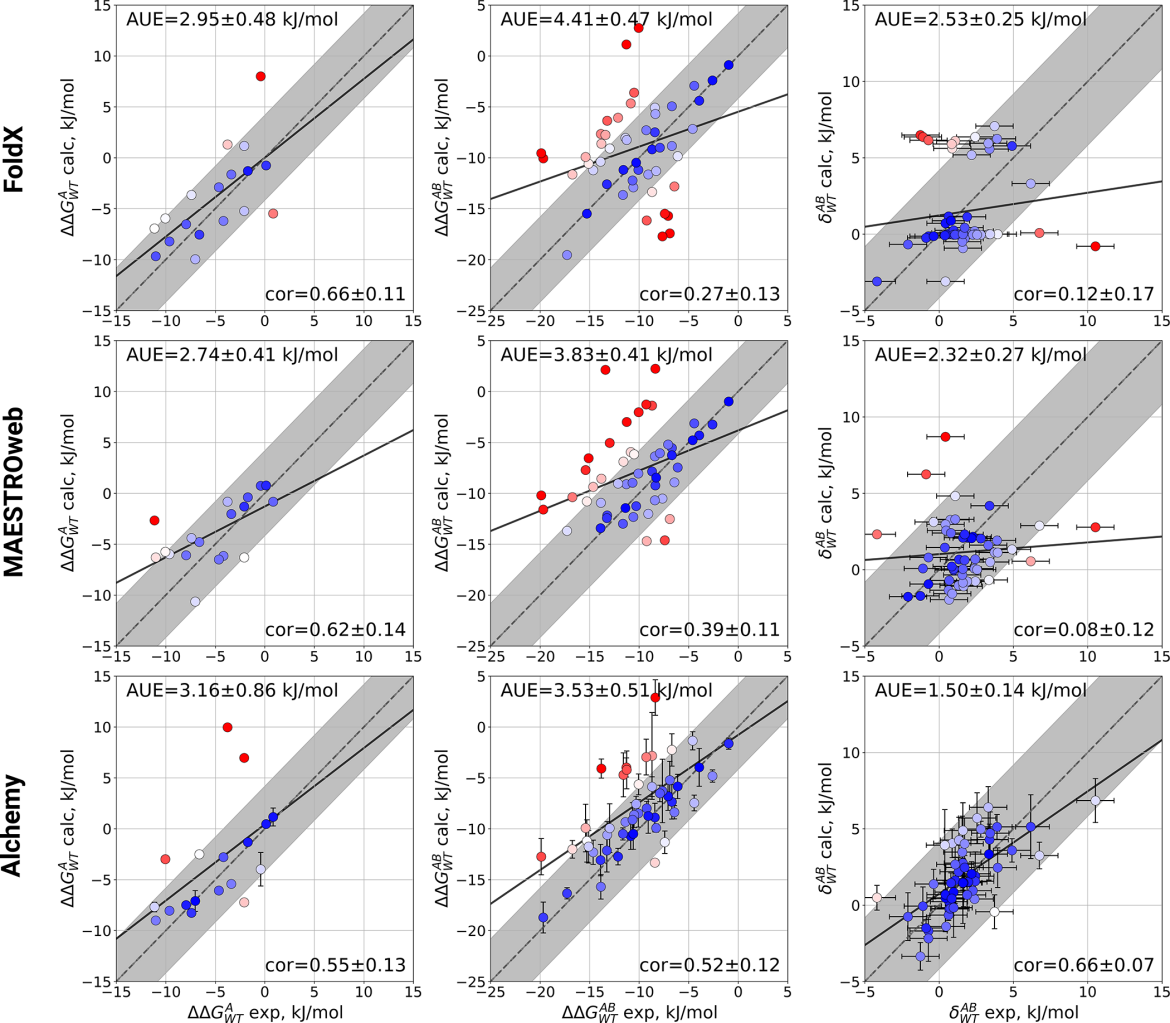
Correlation plots of the free energy calculations for staphyloccocal nuclease mutations with FoldX, MAESTROweb, and the alchemical free energy calculation protocol. Shown are the 18 single mutations (first column), the 52 double mutations (second column) and the corresponding nonadditivities (third column), with experimental data taken from ref (6). The shaded area marks the ±1 kcal/mol (4.184 kJ/mol) difference between calculation and experiment, while the solid line shows linear regression of the data. In each plot the average unsigned error (AUE) and Pearson correlation coefficient are shown in the top left and bottom right corner, respectively.

For the double mutations (Figure 2, middle column), however, correlation with the experiment drastically drops for FoldX and MAESTROweb. The alchemical protocol exhibits the lowest AUE, i.e. highest accuracy, and largest correlation with experiment for this data set.

Of the three methods the alchemical protocol is the only approach to accurately reproduce the nonadditivity with a low AUE and the highest correlation with experiment found (Figure 2, right column). The prediction accuracy is retained across a range of inter-residue distances for the considered mutations (Figures S2,S3); that is, nonadditivities of distant residue pairs are captured as well as those of the proximal residues. This may be because the alchemical approach is not trained on a given data set, but is rather purely physics-based. The nonadditivities in the alchemical approach are calculated under the explicit assumption that the effects are additive in the unfolded state. Hence, unfolded state calculations cancel out from the thermodynamic cycle, removing errors associated with unfolded state contributions. The obtained accuracy for the computed nonadditivities appears to justify this approximation.

We have also benchmarked the computational methods on two other proteins by predicting nonadditivities in barnase and myoglobin (SI). These additional tests, however, do not allow us to draw statistically significant conclusions about the performance of the methods and mainly shed light on the challenges associated with prediction of nonadditive effects. For the case of myoglobin (Figures S4,S5), the limited dynamic range of the experimentally measured nonadditivities allows each of the computational methods to achieve good overall agreement with the measurements (AUE up to 2.7 ± 0.5 kJ/mol). These seemingly accurate predictions, however, are obtained despite that all the approaches failed to detect any nonadditive effects. Barnase calculations showcase a different scenario (Figures S6,S7), where the experimental uncertainties are considerable (due to different free energy estimation strategies as discussed in the SI text). The large experimental uncertainties make it difficult to interpret computational predictions.

The studied mutational pairs cover a large variety in terms of inter-residue distances and type of introduced perturbations. A strong coupling is expected for pairs in close spatial contact due to direct interactions between the residues, but can still be persistent for remote mutations. For example, the mutation pair I72V+Y113A is more than 25 Å apart but displays a nonadditivity of 3.34 kJ/mol in the experimental study. From the tested computational approaches only the alchemical free energy calculation protocol is capable of reproducing the nonadditive behavior of this distant pair (SI Table S4), while additive free energies are predicted by the other two approaches. Thus, we turned to investigating long-range thermodynamic couplings on the basis of the alchemical free energy calculation protocol.

In the analysis of the physical mechanisms that underlie mutational nonadditivities, we first concentrate on the L37A/G79S mutation, and explore the effects of external mutations on the nonadditive character of this residue pair. The external mutation refers to a mutation which does not involve either of the residues L37 or G79. Experimentally, Green and Shortle created triple mutations of staphylococcal nuclease for the mutation pair L37A/G79S.^6^ The coupling between L37A and G79S experimentally amounts to 10.51 kJ/mol (Table 1), the largest nonadditivity of the whole data set. The alchemical approach was able to capture the strong nonadditive effect for this mutation pair (6.85 ± 1.44 kJ/mol). A larger than average deviation from the experimental measurement in this case likely occurs due to glycine involving mutation: since glycine does not restrict the backbone motion in the same way as other canonical amino acids, it presents a larger sampling challenge. The introduction of external mutations to this pair yielded cases such as the L37A+G79S+N118D mutant, in which the resulting free energy of unfolding was indistinguishable from the L37A single mutation leaving the other two mutations completely masked in this triple mutation variant.

**Table 1:**
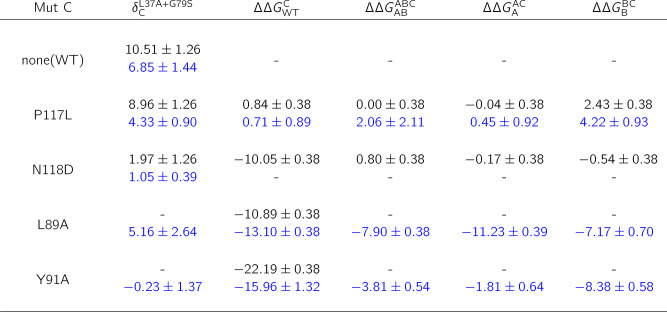
Nonadditivities and Individual Free Energy Contributions for External Mutations Affecting the L37A+G79S Mutation Pair^a^

a δ_C_^L37A+G79S^ corresponds to the L37A+G79S nonadditivity when mutation *C* is introduced in the protein. The experimental results shown in black have been deduced from the corresponding triple mutation free energies provided in ref (6) and ref (27), while simulation results for charge-conserving mutations are displayed in blue. All values are provided in kJ/mol.

For the strongly nonadditive L37A+G79S pair, the effects of the third mutation are summarized in Table 1 for a set of nearby mutations with their position in the 1STN crystal structure displayed in Figure 3a. In addition to the resulting thermodynamic coupling of the L37A+G79S mutation pair under the effect of the third mutation, the individual free energy contribution of every mutational state of the original double mutant cycle are shown. Such a breakdown of nonadditivity changes into independent free energy contributions allows pin-pointing the states that are most affected by the mutation.

**Figure 3:**
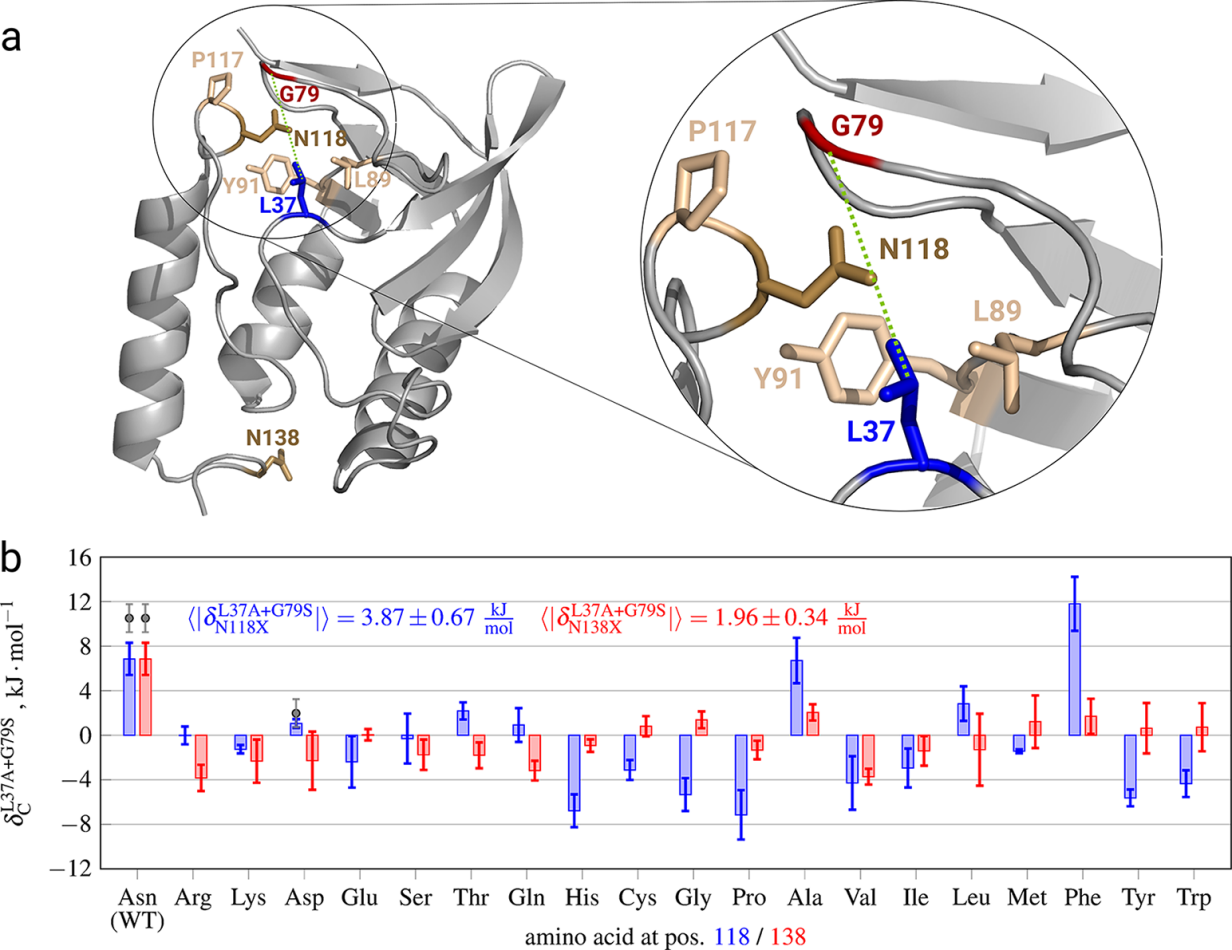
(a) Locations of the Leu37 (blue) and Gly79 (red) amino acids in the staphylococcal nuclease wild type crystal structure (1STN). The other colored residues were mutated to probe their influence on the thermodynamic coupling of the L37A+G79S mutation pair. (b) Mutational scan of the proximal Asn118 (blue) and distant Asn138 (red) amino acids probing their effect on the thermodynamic coupling of the nonadditive L37A+G79S. Gray dots display experimental data taken from ref (6). The unsigned nonadditivities averaged over all mutations and the respective standard errors are shown within the plot.

The introduction of an additional mutation, e.g. P117L (Table 1), does not only have little effect on the strongly nonadditive character of the L37A+G79S mutation pair, but also yields only small free energy changes when introduced to the different states of the double mutant cycle. The computation also captures L37A+G79S nonadditivity for the P117L variant: similarly as for the WT protein, the estimated nonadditive effect is lower when compared to the experimental measurement. A very different behavior is observed for N118D as a third mutation (for this mutant we computed nonadditivity only based on the equation in Figure 1b, thus no individual ΔΔ*G* values are provided in Table 1 to avoid direct introduction of charge perturbing mutations). The L37A+G79S pair is almost perfectly additive if N188D mutation is used as the new reference state. The effect can also be traced down by looking at the individual free energy changes. While N118D has almost no effect when introduced to the single and double mutant variants of the L37A+G79S pair, the change is significant when mutating the wild type protein.

The external mutation L89A kept the nonadditivity of the L37A+G79S pair almost unchanged, but it had a significant impact on the resulting free energies when introduced to each of the mutational states separately. Another mutation, Y91A, was found to erase the nonadditive character of the original pair. In this case, however, the origin of the interaction cannot be attributed solely to the effect on the wild type state of the double mutant cycle as the free energy change caused by Y91A is substantial when applied to the G79S mutational state as well.

The full picture of the effect of an external third mutation on a particular residue coupling can be obtained by computationally scanning all possible mutations for the external residue. We have performed such a scan to investigate changes in the L37A+G79S coupling upon mutation of a proximal N118 and distal N138 (Figure 3b).

The scan revealed previously unknown mutations that can erase the thermodynamic coupling similarly to N118D. Interestingly, there are also mutations found that keep the thermodynamic coupling between the L37A+G79S mutations intact (N118A), increase its absolute value (N118F), or even invert its sign with a remaining strong coupling (e.g., N118H, N118G, N118P). For mutations at the distant position 138 no such strong couplings are found, and also the average nonadditivities are significantly lower than for the N118 mutations. The largest identified coupling in this case, however, marks asparagine N138 as an important amino acid required for the thermodynamic coupling of the L37A+G79S mutational pair even though it is located at ∼20 Å from L37.

For a mutation pair that is almost perfectly additive (L37A+Y113A) given the wild type reference state, mutations at positions 118 and 138 also have a variety of effects on the corresponding nonadditivities (SI Figure S8). The L37A+Y113A coupling is sensitive to mutations at both proximal N118 and distal N138 locations.

As the alchemical predictions are based on the rigorous physical model, the underlying trajectories of protein dynamics provide mechanistic details of the residue networks affecting nonadditivities. In Figure 4 we explain how the nonadditivity arises in the case of L37A+G79S mutation and how it is further modulated by N118 and N138 mutations. The residue packing in the WT protein forms an ordered structure which remains stable in the MD simulation. The residue N118 interacts with G79 via a hydrogen bond to the backbone. Also, N118 has favorable interaction energy with L37. Mutation L37A removes both interactions (inset in the Figure 4, upper panel), thus having a destabilizing effect. Mutating G79S is even more destabilizing: N118 starts forming transient contacts with serine, but cannot retain a stable fold of the loop. The double mutant L37A+G79S becomes more stable, because N118 is brought into the conformation similar to that of the single L37A mutation.

**Figure 4:**
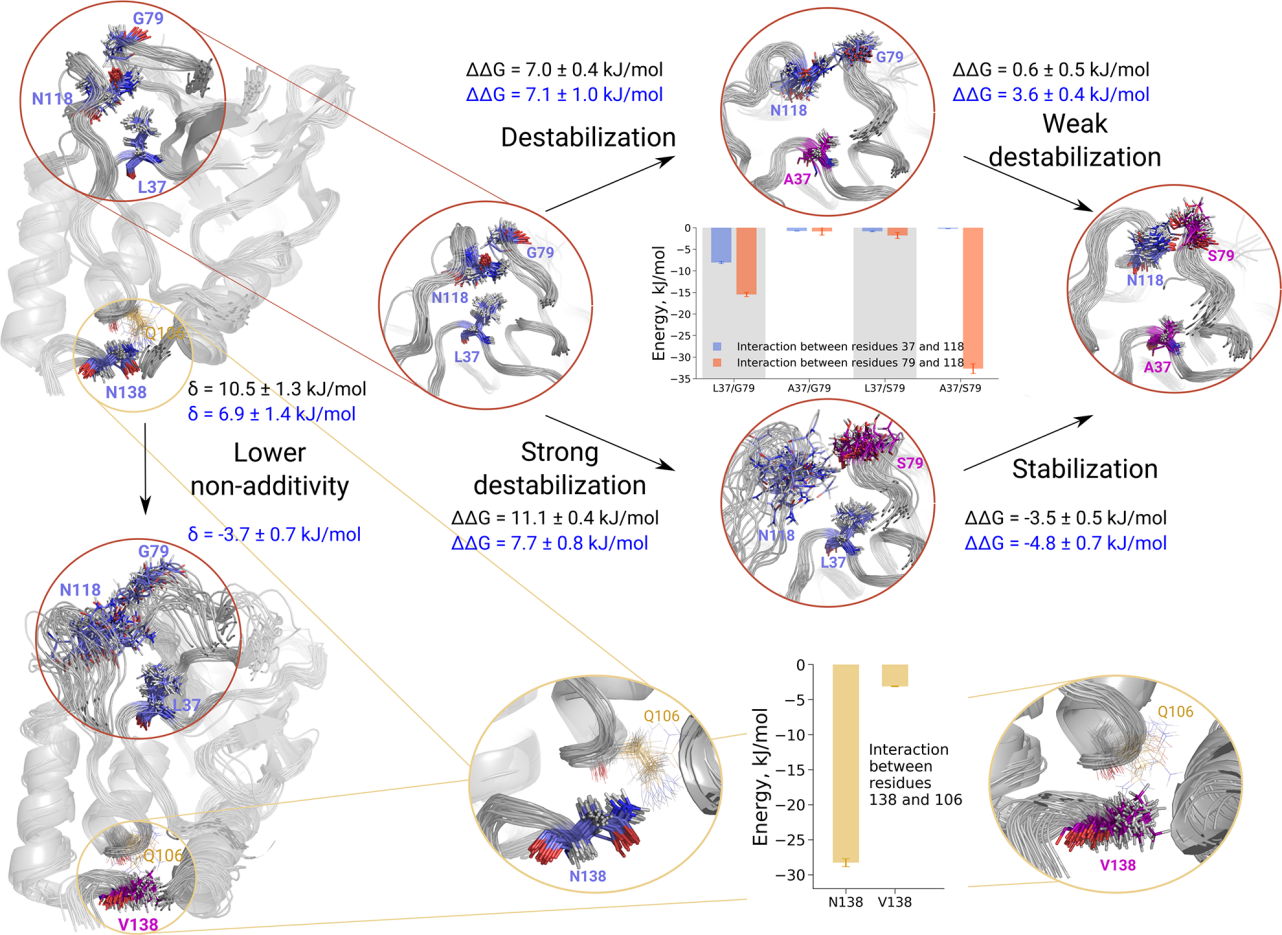
Network of residues affecting L37A+G79S nonadditivity. The upper panel illustrates the origin of nonadditivity: the mutations L37A and G79S individually are destabilizing, while introduction of both mutations together is more favorable. This effect is further explained by the interaction energy calculations in the inset figure. The lower panel explains how a distant N138V mutation alters the overall stability of the protein’s loop harboring N118 residue, which in turn modulates L37A+G79S nonadditivity. The experimentally measured changes in stability and nonadditivities are shown in black, computed values are in blue.

Interestingly, a distant mutation (N138V Figure 4, lower panel) can change the whole nonadditivity interplay. In the WT protein N138 forms a hydrogen bond with the backbone of Q106. Mutating N138V disrupts this interaction, and in turn the whole helix-loop with N118 is distorted. This way, the whole subtle mechanism of L37A+G79S nonadditivity is perturbed.

The systematic mutational scans (Figure 3) and the residue network analysis (Figure 4) showcase that the thermodynamic coupling of a mutant pair is highly case specific. While for a given position the coupling can be negligible for some amino acids, other residues may exhibit strong nonadditivities. A coupling between a pair of amino acids can be controlled by a third mutation, which does not need to be in the direct vicinity of either residue of the pair. Being able to predict and quantify such amino acid specific effects on couplings presents a new perspective to interpreting allosteric networks. The network cannot be based on two-body correlations between residue positions only, but rather the correlations need to be conditioned on all the residues respecting their type. Realizing this additional level of complexity practically, could be the next essential step in advancing protein design, similarly as considering intraresidue correlations by AlphaFold has changed the landscape of protein fold prediction.^28^

To sum up, the first-principles based calculations are able to capture coupling effects between protein residues. This enables access to exploring physical mechanisms underlying long-range interactions between amino acids. In the future this approach may allow construction of entire allosteric networks based on the rigorous free energy calculations.

## Supporting Information Available

Computational details, derivation of the equation for nonadditivities, analysis of the SNase nonadditivity prediction by the alchemical method for different inter-residue ranges, investigation of mutation effects on the free energy changes and nonadditivities in myoglobin and barnase, scan probing the effects of a nearby and distant amino acid mutations on the nonadditivities of the L37A+Y113A pair, table summarizing the calculated nonadditivities when following two different calculation strategies, tables with the experimental and calculated free energy differences and nonadditivities for SNase, myoglobin, and barnase (PDF)
