# Supplementary material for: One Plus One Makes Three: Triangular Coupling of Correlated Amino Acid Mutations
Source: J Phys Chem Lett. 2021 Mar 24;12(12):3195–201. doi: 10.1021/acs.jpclett.1c00380 (PMC8041375; doi:10.1021/acs.jpclett.1c00380)
Supplement: Supplementary file 1 — jz1c00380_si_001.pdf [file jz1c00380_si_001.pdf]

Supporting Information: One Plus One Makes Three: Triangular Coupling of Correlated Amino Acid Mutations

Martin Werner†, Vytautas Gapsys† and Bert L. de Groot*

Computational Biomolecular Dynamics Group, Max-Planck Institute for Biophysical Chemistry, Am Fassberg 11, 37077, Göttingen, Germany. Tel.: +(49)551-2012308, Fax: +(49)551-2012302. *email: bgroot@gwdg.de

† These authors contributed equally.

Computational Details Simulations of the alchemical free energy protocol were carried out in Gromacs 2016 for staphyloccocal nuclease and Gromacs 2018 for myoglobin and barnase.(1, 2) Staphyloccocal nuclease mutants were created starting from the 1STN (3) PDB structure of wild type staphyloccocal nuclease using FoldX. For myoglobin and barnase the structures 1BZ6 (4) and 1A2P (5) were used, respectively. Each protein was placed in a TIP3P (6) water box. For staphyloccocal nuclease and barnase, NaCl ions (7) were added to neutralize the system and reach concentration of 0.15 mol/L. For myoglobin simulations we used 0.1 mol/L KCl ion concentration. The force field of choice was AMBER99sb*ILDN (8–10) which provided accurate results in previous alchemical free energy studies.(11, 12) For myoglobin simulations, heme was parameterized using the tool MCPB (13), AmberTools (14) and Gaussian (15). In addition, myoglobin simulations were also performed with the Charmm36m (16) force field.

Staphyloccocal nuclease simulations were performed at 293 K temperature, while myoglobin and barnase were simulated at 298 K. The temperature was controlled by means of the velocity rescaling thermostat (17) with the time constant of 0.1 ps. The pressure was kept at 1 bar using the Parrinello-Rahman barostat (18) with the time constant of 5 ps. The electrostatic interactions were treated by means of PME (19, 20) with the real space cutoff of 1.2 nm and the grid spacing of 0.12 nm. The van der Waals interactions were shifted to zero at the cutoff of 1.2 nm. An analytical dispersion correction for the energy and pressure was applied. Bonds involving hydrogen atoms were constrained by the LINCS (21) algorithm of the order 6. For the alchemical transitions, the non-bonded interactions were softened (22).

A pre-equilibration of 20 ns was carried out. This equilibration was used as a starting point for 30 independent runs of 20 ns length for the wild type and each mutant. From each run 100 non-equilibrium transitions of 100 ps length were spawned using a local development version of pmx.(23) Work values from the forward and backward transitions were collected using thermodynamic integration (TI) to estimate the corresponding free energy with Bennett's acceptance ratio (BAR) as a maximum likelihood estimator(24) relying on the Crooks Fluctuation Theorem.(25) For the thermodynamic cycles of triple mutants (main text Table 1), cycle closure correction was applied.(26)

The calculated and experimentally measured values were compared by means of Pearson correlation coefficient and average unsigned error (AUE), where the latter is defined as:

$$\mathrm{AUE} = \frac{\sum_{i=1}^{N} |x_i^{calc} - x_i^{exp}|}{N} \tag{S1}$$

Here, N is the number of observations, x_i^{calc} and x_i^{exp} are the calculated and experimental values, respectively.

Detailed description of the thermodynamic cycles In alchemical free energy approaches for the calculation of protein stability a thermodynamic cycle as shown in the main text Figure 1a is constructed. The alchemical pathway allows for the calculation of the free energy differences via contributions of the folded $\Delta G_{(\mathrm{f})}$ and the unfolded $\Delta G_{(\mathrm{u})}$ state rather than via the individual free energies of unfolding of the wild type $\Delta G_{(\mathrm{WT})}$ and the mutant $\Delta G_{(\mathrm{A})}$. This yields access to the free energy of unfolding upon amino acid mutation without the actual simulation of an unfolding event in either mutational state which would represent a computationally demanding task.

The unfolded protein state is represented by a capped tripeptide recovering the original sequence environment. This was found to be a more accurate representation than a simple tripeptide chain without information on the protein sequence.(27)

For double mutants an additional thermodynamic cycle can be constructed as shown in the main text Figure 1b. This double mutant cycle contains the double and individual single mutational states and displays three different pathways for the calculation of the free energy upon double mutation. As the free energy is a state function, these pathways are constructed to exactly match each other. This is exploited in the alchemical free energy approach by calculation of the double mutation free energy via all three pathways to obtain an average estimate and the corresponding standard error. The same is true for the nonadditivity of the free energies. By substitution of the different free energy pathways for the double mutation into equation in the main text Figure 1b one obtains also three different possibilities for the calculation of $\delta_{\mathrm{WT}}^{\mathrm{AB}}$. It should be noted that for nonadditivities regarding the free energies of unfolding the contributions of the unfolded state vanish, as its representation assumes the free energy changes to be additive within this state. Thus nonadditivities are solely calculated from folded state contributions within this alchemical free energy approach. A procedure as shown for the double mutant free energy in form of the calculation via all three different pathways with the average estimate and standard error is also followed for the nonadditivity.

FoldX Setup For the free energy calculation with FoldX the 1STN structure of staphylococcal nuclease was pre-optimized using the *RepairPDB* command as

```
foldx --command=RepairPDB --pdb=1stn.pdb
```

Each mutational state A was constructed with the *BuildModel* command by using

```
foldx --command=BuildModel --pdb=1stn_Repair.pdb --mutant-file=individual_list.txt
```

The free energy of unfolding of the individual mutant was calculated with the *Stability* command as

```
foldx --command=Stability --pdb=1stn_Repair_1.pdb
```

and finally combined with the result for the wild type to obtain the corresponding $\Delta\Delta G^{\mathrm{A}}_{\mathrm{WT}}$. The free energy changes of double mutants were calculated accordingly and combined with the individual results for the calculation of nonadditivities.

MaestroWeb Setup The MaestroWeb websever was contacted via

https://pbwww.che.sbg.ac.at/maestro/web.

Projects of individual and combined amino acid mutations were created for the 1STN crystal structure at pH=7. The free energy changes were extracted and combined for the calculation of the corresponding nonadditivities.

FoldX and MaestroWeb calculations for myoglobin and barnase followed the same procedures as described for staphyloccocal nuclease, except for setting pH=9.6 for the case of myoglobin to match the experimental conditions. (29, 30) The starting structures 1BZ6 and 1A2P were used for myoglobin and barnase, respectively.

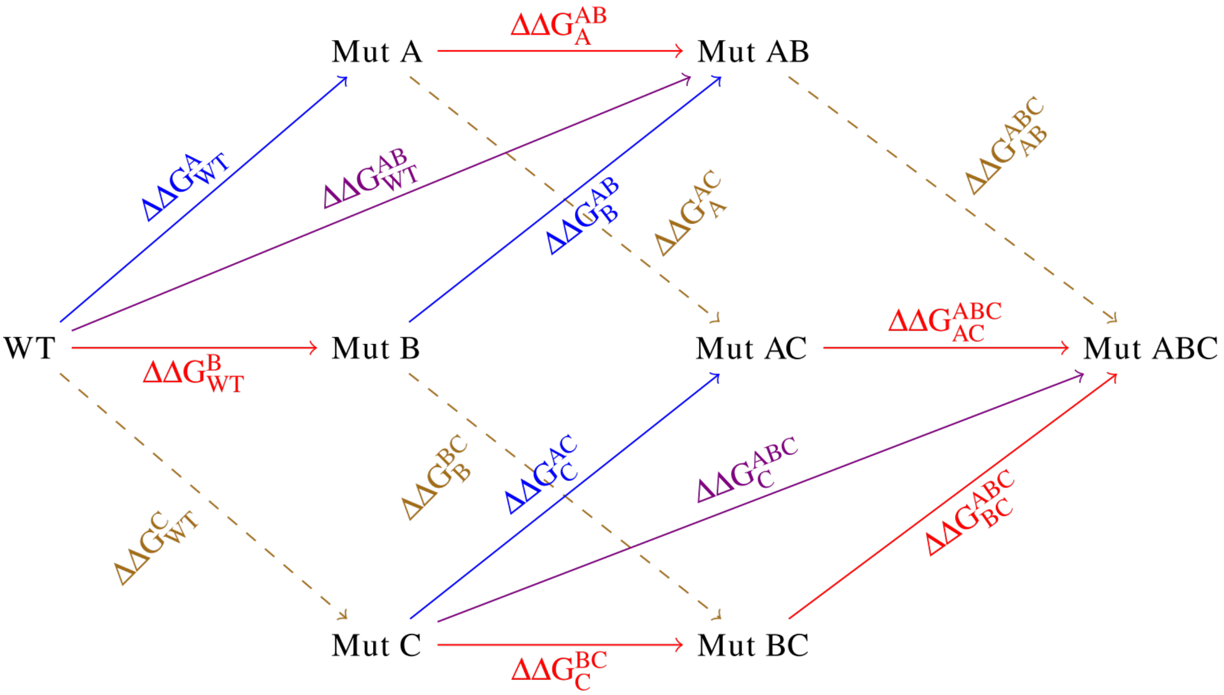

$$\Delta\Delta G_{C}^{ABC} = \Delta\Delta G_{WT}^{AB} + \Delta\Delta G_{AB}^{ABC} - \Delta\Delta G_{WT}^{C}$$
$$\Delta\Delta G_{C}^{AC} = \Delta\Delta G_{WT}^{A} + \Delta\Delta G_{A}^{AC} - \Delta\Delta G_{WT}^{C}$$
$$\Delta\Delta G_{C}^{BC} = \Delta\Delta G_{WT}^{B} + \Delta\Delta G_{B}^{BC} - \Delta\Delta G_{WT}^{C}$$
$$\delta_{WT}^{AB} = \Delta\Delta G_{WT}^{AB} - \left(\Delta\Delta G_{WT}^{A} + \Delta\Delta G_{WT}^{B}\right)$$
$$\delta_{C}^{AB} = \Delta\Delta G_{C}^{ABC} - \left(\Delta\Delta G_{C}^{AC} + \Delta\Delta G_{C}^{BC}\right)$$

$$\begin{aligned}\delta_{C}^{AB} &= \Delta\Delta G_{C}^{ABC} - \left(\Delta\Delta G_{C}^{AC} + \Delta\Delta G_{C}^{BC}\right)\\ &= \Delta\Delta G_{WT}^{AB} + \Delta\Delta G_{AB}^{ABC} - \Delta\Delta G_{WT}^{C} - \left(\Delta\Delta G_{WT}^{A} + \Delta\Delta G_{A}^{AC} - \Delta\Delta G_{WT}^{C} + \Delta\Delta G_{WT}^{B} + \Delta\Delta G_{B}^{BC} - \Delta\Delta G_{WT}^{C}\right)\\ &= \underbrace{\Delta\Delta G_{WT}^{AB} - \left(\Delta\Delta G_{WT}^{A} + \Delta\Delta G_{WT}^{B}\right)}_{\delta_{WT}^{AB}} + \Delta\Delta G_{AB}^{ABC} + \Delta\Delta G_{WT}^{C} - \left(\Delta\Delta G_{A}^{AC} + \Delta\Delta G_{B}^{BC}\right)\end{aligned}$$

$$\delta_{C}^{AB} = \delta_{WT}^{AB} + \Delta\Delta G_{WT}^{C} + \Delta\Delta G_{AB}^{ABC} - \left(\Delta\Delta G_{A}^{AC} + \Delta\Delta G_{B}^{BC}\right)$$

Figure S1: Derivation of the equation in the main text Figure 1c, where δ_{C}^{AB} is expressed by using contributions from the WT protein nonadditivity and single mutations of different reference states only.

Staphylococcal nuclease

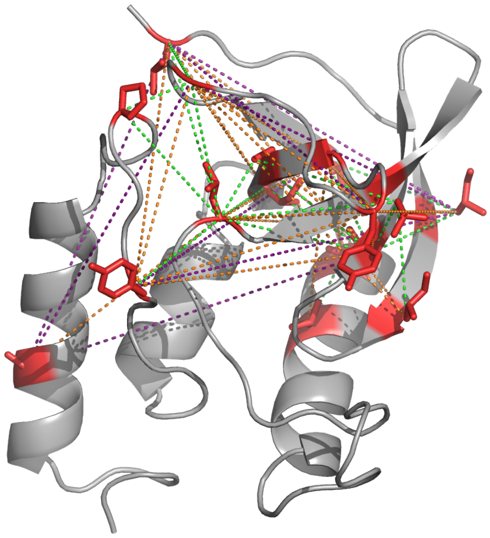

Figure S2: Staphylococcal nuclease structure (pdb id 1STN (3)) with the investigated mutations highlighted in color. The residue pairs whose nonadditivities were calculated are connected with the dashed lines which are color coded by the minimal inter-residue distance considering heavy atoms only: green - closer than 1 nm, orange - between 1 and 2 nm, purple - further than 2 nm.

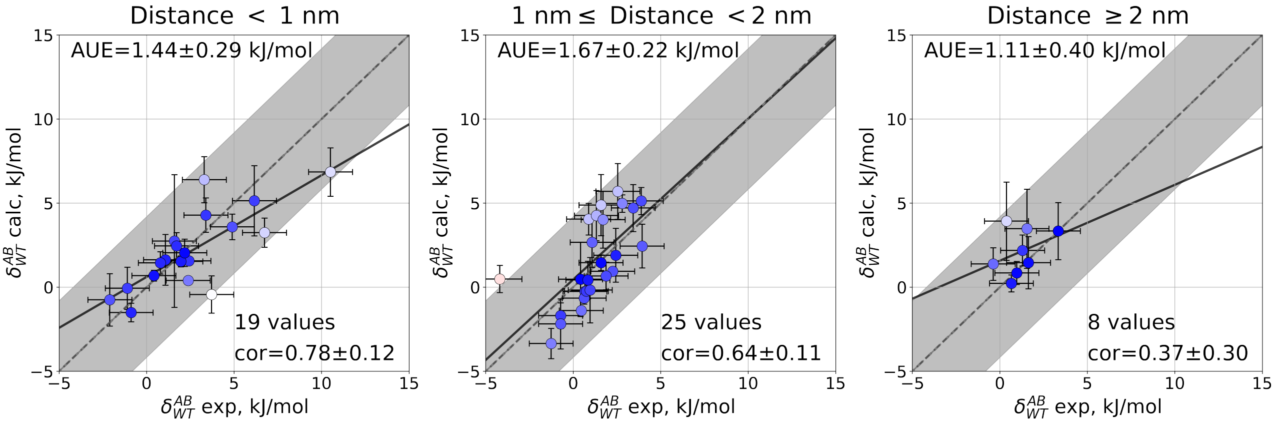

Figure S3: Correlation plots of the free energy calculations for staphylococcal nuclease mutations with the alchemical free energy protocol. The same data as in the main text Figure 2 is depicted by splitting the set into subsets based on the inter-residue distances between the residue pairs for which nonadditivities were calculated. Experimental data taken from (28). The shaded area marks the ±1 kcal/mol (4.184 kJ/mol) difference between calculation and experiment, while the solid line shows linear regression of the data. In each plot the average unsigned error (AUE) and Pearson correlation coefficient are shown in the top left and bottom right corner, respectively. In the left panel, nonadditivities of the residues that are closer than 1 nm apart (considering minimal distance between any heavy atom) are shown. Middle panel, inter-residue distance between 1 and 2 nm. Right panel, inter-residue distance larger than 2 nm. The dynamic range of the experimentally measured nonadditivities decreases for the residues that are further apart, i.e. the effect of mutating two distant residues is more additive in nature. The computational prediction performs well in each of the distance ranges in terms of AUE. The Pearson correlation between the calculated and experimental values decreases for the most distant residue pairs (right panel), but that is likely an effect of a small dataset and a narrow dynamic range of nonadditivities, since the AUE for this subset shows a good agreement between computation and experiment (1.11±0.40 kJ/mol).

Myoglobin investigation

We have calculated 8 myoglobin nonadditivities and compared them to the experimentally measured values constructed from the folding free energy differences reported in (29, 30). The mutated residues are depicted in Figure S4, while the calculation results are in Figure S5. The alchemical calculations based on Amber99sb*ILDN force field performed remarkably well in predicting the free energy changes upon single and double mutations. MAESTROweb performed comparably well to the alchemical approach, while FoldX had a considerably lower accuracy. Interestingly, all the considered methods perform equivalently in predicting nonadditivities: the computational predictions agree that the considered mutations should be mainly of additive nature. To further verify this prediction, we have performed alchemical calculations with the Charmm36m force field, which yielded the result consistent with the other calculations.

Overall, for the myoglobin nonadditivity data, the computational methods perform similarly and the narrow dynamic range of the data set does not allow making statistically reliable conclusions about the differences between the methods. The consistent predictions from the conceptually different approaches (including alchemical calculations with two force fields) suggest that there might be a common underlying reason for the discrepancies when comparing to experiment. One of the potential sources of such disparities could be the initial myoglobin structure used for computations and the experimental conditions under which the unfolding free energies were measured. For the computations we relied on a high resolution X-ray structure (1.2 Å, 1BZ6) where myoglobin was resolved in its aquomet form at pH of 6 (4). The unfolding experiments, however, were performed in the presence of 0.5 mol/L KCN concentration at the pH of 9.6, thus stabilizing the met form of myoglobin (30). A further in depth investigation into the different myoglobin forms would be necessary to identify the dependencies of the nonadditivities on the heme state and myoglobin conformation.

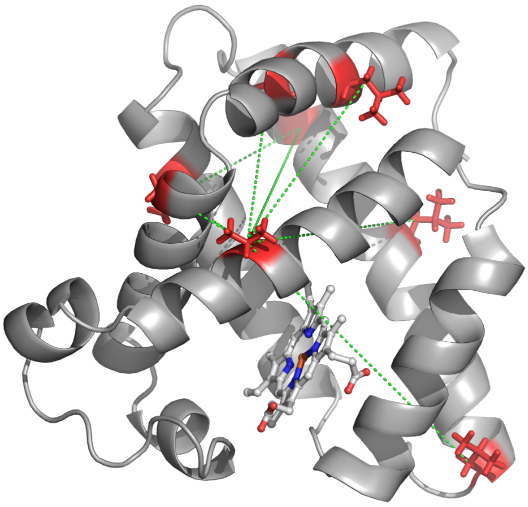

Figure S4: Myoglobin structure (pdb id 1BZ6 (4)) with the investigated mutations highlighted in color. The residue pairs whose nonadditivities were calculated are connected with the dashed lines.

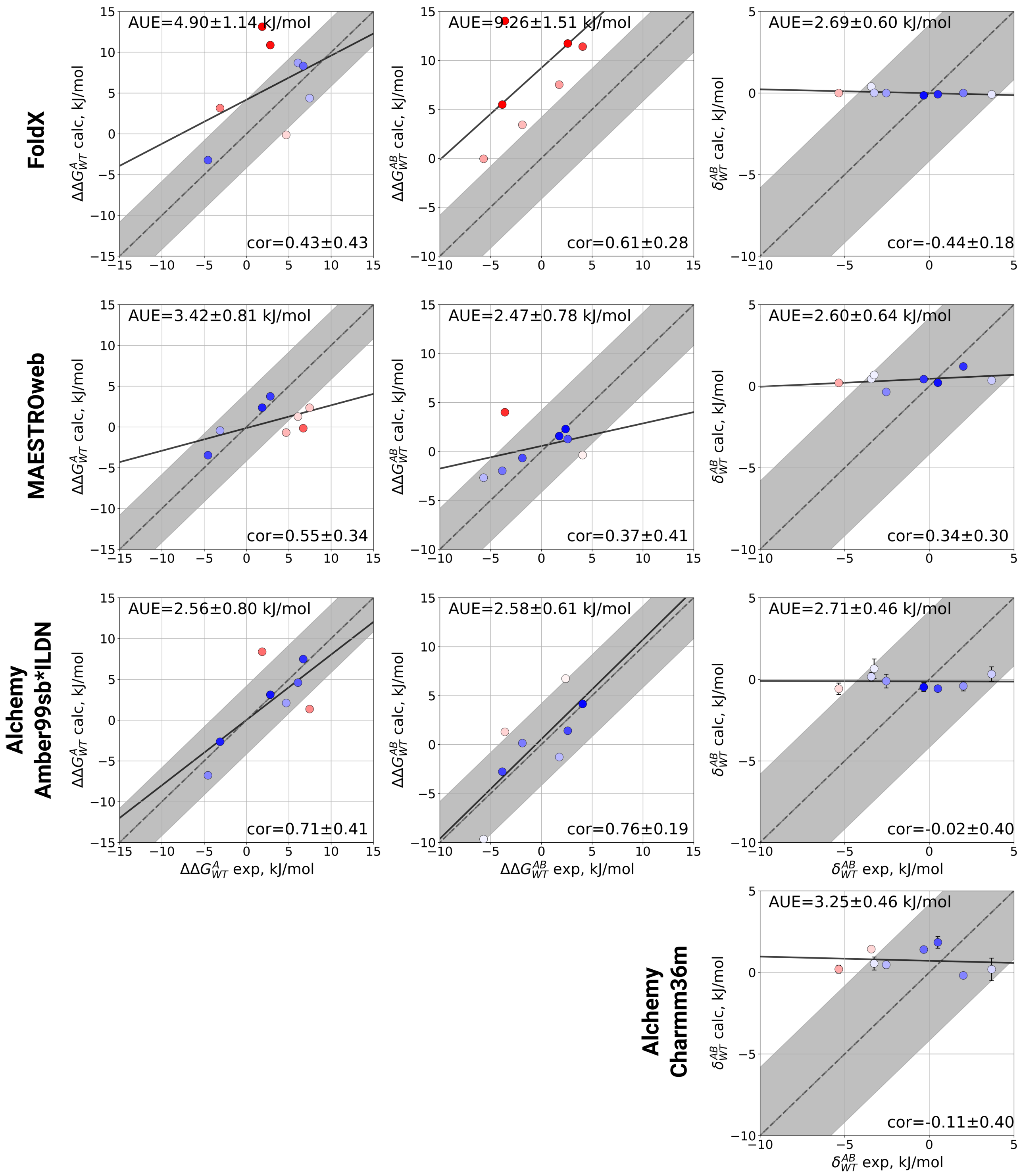

Figure S5: Correlation plots of the free energy calculations for myoglobin mutations with FoldX, MAESTROweb and the alchemical free energy protocol. Shown are the 8 single mutations (first column) with the experimental data from (29), the 8 double mutations (second column) and the corresponding nonadditivities (third column), with experimental data taken from (30). The shaded area marks the ±1 kcal/mol (4.184 kJ/mol) difference between calculation and experiment, while the solid line shows linear regression of the data. In each plot the average unsigned error (AUE) and Pearson correlation coefficient are shown in the top left and bottom right corner, respectively.

Barnase investigation

The estimated free energy accuracy for the single mutations in barnase is comparable for all the investigated computational methods showing no significant differences (Fig S7). The prediction accuracy decreases for the double mutants, where MAESTROweb shows the most substantial deviation from the experimentally measured values. Interestingly, all the methods perform comparably in predicting nonadditivities, with the AUE ranging from 2.5 ± 0.2 kJ/mol for FoldX to 3.1 ± 0.2 kJ/mol for the alchemical calculations. Overall, it appears that each computational method is capable to accurately predict free energy differences and nonadditivities for a subset of experimental measurements. Notably, the quantification in Figure S7 in a number of cases uses multiple experimentally measured values for the same nonadditivity or free energy difference reflecting the uncertainty in the $\Delta\Delta G$ estimation from the experimental data. To better understand the origin of this uncertainty, we describe in more detail the experimental methods used to obtain the unfolding free energy differences and how they result in a wide range of nonadditivity estimates.

Experimentally, barnase unfolding free energies have been measured by means of urea induced unfolding applying two different methods for quantification. In the first approach $\Delta G_u^{H_2O}$, free energy of unfolding in water, is obtained by titrating barnase with urea, monitoring changes in the fluorescence spectrum to estimate unfolding free energy ΔG_u and fitting the curve (31):

$$\Delta G_u = \Delta G_u^{H_2O} - m[urea] \tag{S2}$$

In this approach, the unfolding free energy in water will be sensitive to even small uncertainties in fitting the slope m.

An alternative method makes use of an observation that the unfolding free energy can be estimated in a highly reproducible manner at the denaturant concentration where 50% of unfolding is observed ($[urea]50\%$). Namely, $\Delta G_u^{[urea]50\%}$ can be used to precisely determine the difference in the unfolding free energies $\Delta\Delta G_u^{[urea]50\%}$ from the average value $\langle m \rangle$ and the difference in $[urea]50\%$ concentration for the wild type and mutant protein (31)

$$\Delta\Delta G_u^{[urea]50\%} = \langle m \rangle \Delta[urea]50\% \tag{S3}$$

Alternatively, the $\Delta\Delta G_u^{[urea]50\%}$ can be obtained directly, by titrating and measuring $\Delta\Delta G_u^{[urea]x}$ at different urea concentrations. The estimation of $\Delta\Delta G_u^{H_2O}$ based on the $\Delta\Delta G_u^{[urea]50\%}$, however, needs to include the transfer free energies of individual side-chains from water to urea (31):

$$\Delta\Delta G_u^{[urea]50\%} = \Delta\Delta G_u^{H_2O} + \delta g_{tr,i}(WT) - \delta g_{tr,i}(MUT) \tag{S4}$$

The contribution of the transfer free energies $\delta g_{tr,i}$ will depend on the exposure of the side-chain i to the solvent. For the current study, the transfer free energies were approximated by the solubility measurements of amino acids and their derivatives at 4 mol/L urea concentration (32).

We have assembled a set of $\Delta\Delta G_u^{H_2O}$ free energy values for single and double mutants of barnase from a series of publications: (31, 33–38). Combining the free energy measurements from various sources allows obtaining multiple nonadditivities for a single mutation pair (e.g. 64 values for Y13A+Y17A). The uncertainty associated with the nonadditivities can be appreciated from the Figure S7. Taking into account the uncertainties in nonadditivities at least in part explains why the current barnase data set could not provide a robust discrimination of the computational methods.

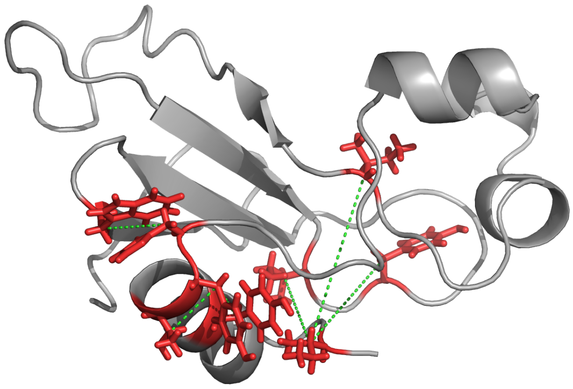

Figure S6: Barnase structure (pdb id 1A2P (5)) with the investigated mutations highlighted in color. The residue pairs whose nonadditivities were calculated are connected with the dashed lines.

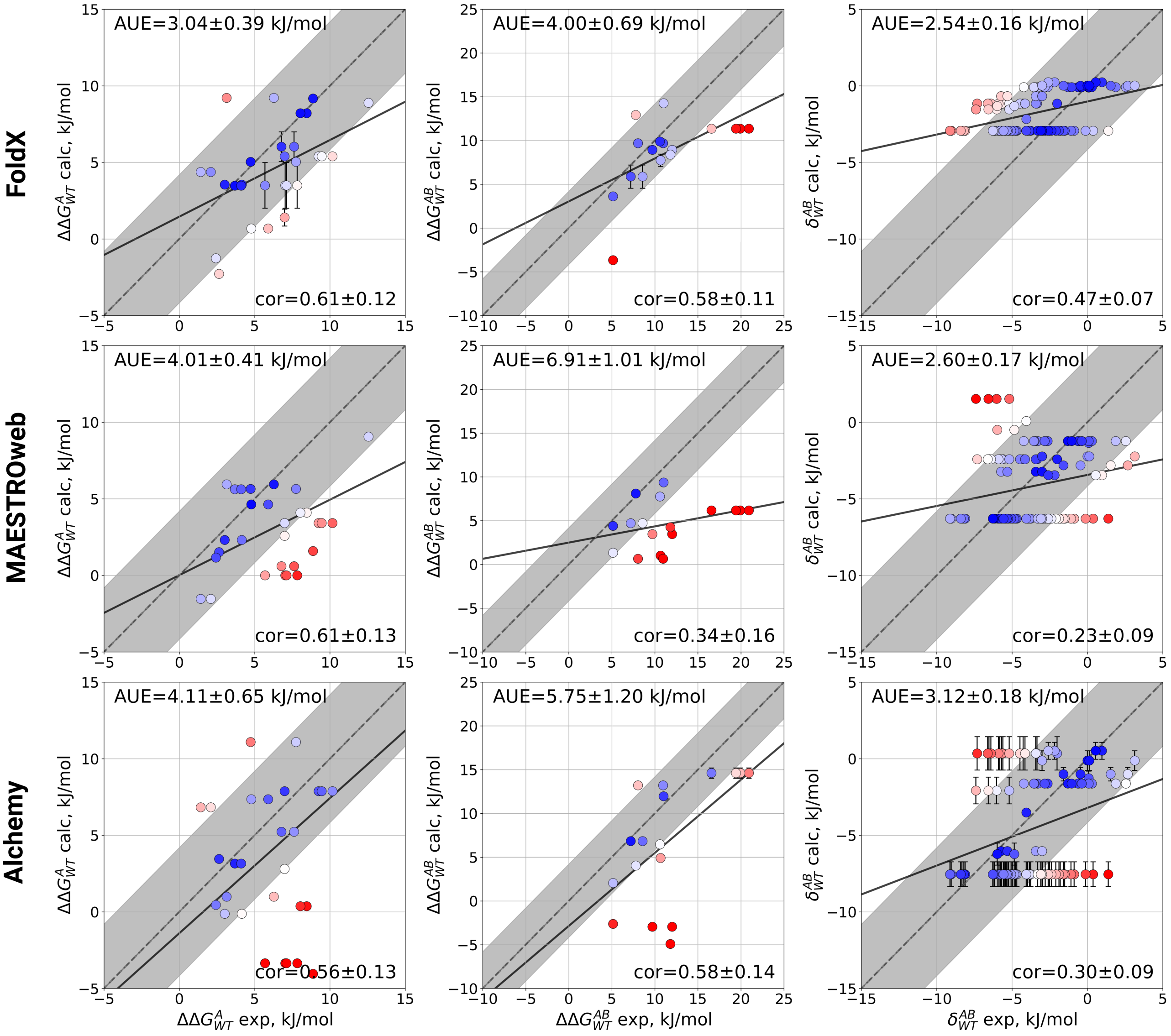

Figure S7: Correlation plots of the free energy calculations for barnase mutations with FoldX, MAESTROweb and the alchemical free energy protocol. Shown are the 15 single mutations (first column), the 11 double mutations (second column) and the corresponding nonadditivities (third column). The sources of the experimental data are summarized in the tables S8 and S9. The shaded area marks the ±1 kcal/mol (4.184 kJ/mol) difference between calculation and experiment, while the solid line shows linear regression of the data. In each plot the average unsigned error (AUE) and Pearson correlation coefficient are shown in the top left and bottom right corner, respectively.

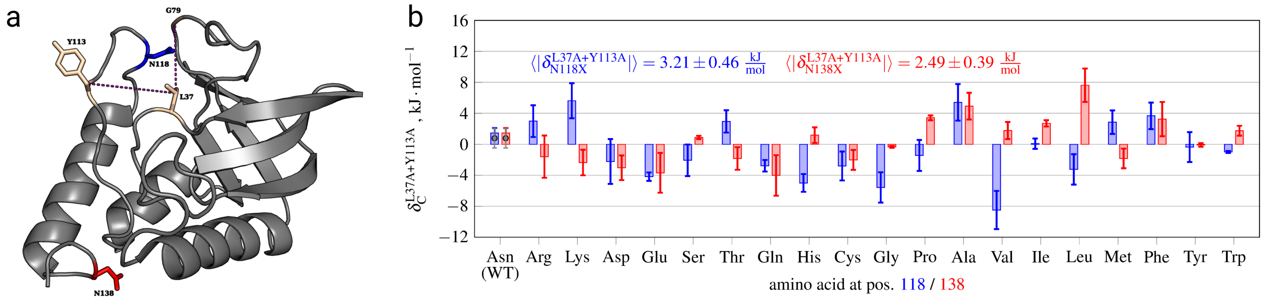

Figure S8: **a** Locations of the amino acids for studying the influence of third mutations on the additive L37A+Y113A mutation pair in the staphylococcal nuclease wild type crystal structure (1STN). The effect was probed for all possible target mutations at the nearby Asn118 amino acid (blue) and the distant Asn138 (red) residue. **b** Mutational scan of the nearby Asn118 (blue) and distant Asn138 (red) amino acids probing their effect on the thermodynamic coupling of the additive L37A+Y113A mutational pair. Gray dots display experimental data taken from (28). The unsigned nonadditivities averaged over all mutations and the respective standard errors are shown within the plot.

Table S1: Comparison of the nonadditivities for external mutations affecting the L37A+G79S mutation pair obtained following two different calculation strategies. The nonadditivities resulting from the corresponding double mutant cycle (Figure 1b) and from following the individual contributions as shown in Figure 1c are provided. The results agree within the statistical uncertainty. All values are given in kJ/mol.

Mut C	$\delta_C^{L37A+G79S}$	
	Figure 1b	Figure 1c
none(WT)	6.85±1.44	-
P117L	4.33±0.90	4.73±3.16
N118D	1.05±0.39	-
L89A	5.16±2.64	4.24±2.71
Y91A	-0.23±1.37	-2.74±3.03

Table S2: Experimental (28, 39) and calculated staphyloccocal nuclease unfolding free energy differences for single mutations ($\Delta\Delta G_{\mathrm{WT}}^{\mathrm{A}}$). All values are provided in kJ/mol.

Mut	exp	FoldX	MAESTROweb	Alchemy	err(Alchemy)
L7A	-6.62	-7.54	-4.78	-2.50	0.26
I15V	-3.39	-1.63	-2.03	-5.41	0.17
I18M	-2.09	1.17	-1.30	6.97	0.22
V23F	-9.63	-8.21	-5.97	-8.05	0.32
T33S	-4.18	-6.20	-6.15	-2.79	0.23
L37A	-7.03	-9.96	-10.63	-7.09	1.02
T62A	-10.05	-5.95	-5.74	-2.98	0.23
V66L	-0.42	8.00	0.74	-3.97	1.65
A69T	-11.01	-9.67	-6.31	-9.01	0.22
I72V	-7.41	-3.64	-4.40	-8.26	0.15
G79S	-11.14	-6.95	-2.66	-7.68	0.47
Y85A	-1.72	-1.30	-0.39	-1.31	0.30
G88V	-3.77	1.30	-0.82	9.97	0.28
A90S	-7.95	-6.53	-6.09	-7.49	0.15
I92V	-2.09	-5.23	-6.30	-7.22	0.16
Y113A	0.13	-0.75	0.77	0.46	0.31
P117L	0.84	-5.48	-0.82	1.16	0.88
A130G	-4.65	-2.89	-6.51	-6.07	0.10

Table S3: Experimental(28) and calculated staphyloccocal nuclease unfolding free energy differences for double mutations ($\Delta\Delta G_{\mathrm{WT}}^{\mathrm{AB}}$). All values are provided in kJ/mol.

Mut	exp	FoldX	MAESTROweb	Alchemy	err(Alchemy)
I15V+I72V	-8.42	-5.07	-6.36	-13.32	0.29
I15V+Y85A	-4.44	-2.93	-3.14	-7.46	0.78
I15V+Y113A	-2.60	-2.39	-3.24	-4.81	0.60
I72V+Y85A	-6.70	-4.94	-5.55	-7.35	1.69
I72V+Y113A	-3.94	-4.40	-4.31	-3.96	1.86
Y85A+Y113A	-0.96	-0.88	-0.98	-1.63	0.55
I18M+T33S	-8.37	-5.69	-9.23	2.89	1.75
I18M+A69T	-13.82	-8.62	-8.56	-4.08	0.94
I18M+A90S	-11.30	1.13	-9.11	-3.97	0.94
T33S+A90S	-13.23	-6.36	-12.17	-10.60	1.32
A69T+A90S	-19.68	-10.05	-11.60	-18.71	1.51
V23F+T33S	-11.39	-8.04	-11.45	-9.33	0.34
V23F+A69T	-16.75	-11.64	-10.37	-12.01	0.87
V23F+A90S	-13.86	-7.66	-10.94	-15.69	1.21
L37A+T33S	-9.25	-16.16	-14.70	-7.98	0.67
L37A+A69T	-17.29	-19.55	-13.68	-16.34	0.55
L37A+A90S	-13.90	-10.38	-13.43	-13.07	1.54
L7A+V23F	-15.28	-15.49	-10.79	-11.19	2.08
L7A+L37A	-6.91	-17.42	-12.53	-5.21	1.84
L7A+G79S	-11.60	-11.18	-6.89	-4.70	2.16
L37A+P117L	-7.08	-15.70	-5.20	-6.79	1.39
T62A+G88V	-13.40	-7.75	2.13	8.10	2.40
V66L+G88V	-8.37	6.20	2.23	7.78	2.04
G79S+P117L	-8.71	-13.36	-1.39	-2.84	4.26
V23F+L37A	-13.27	-12.60	-12.43	-12.13	2.10
V23F+G79S	-19.89	-9.55	-10.21	-12.73	1.78
L37A+G79S	-7.66	-17.71	-10.52	-6.28	3.12
T62A+V66L	-10.05	2.76	-2.04	-5.63	1.02
L7A+I15V	-8.71	-9.17	-7.85	-5.86	0.97
L7A+I72V	-10.09	-11.18	-8.06	-8.48	1.34
L7A+Y85A	-6.70	-8.83	-6.26	-2.25	1.59
L7A+I92V	-6.45	-12.81	-8.92	-8.37	0.65
L7A+Y113A	-4.61	-7.16	-4.79	-1.35	0.89
L7A+A130G	-10.30	-10.47	-11.25	-7.57	0.76
V23F+I15V	-10.84	-4.65	-5.95	-10.73	1.29
V23F+I72V	-12.14	-6.07	-9.05	-12.73	0.81
V23F+Y85A	-10.51	-3.60	-6.16	-8.64	1.25
V23F+I92V	-8.42	-7.49	-10.68	-8.89	1.40
V23F+Y113A	-7.91	-9.00	-6.06	-6.52	0.81
V23F+A130G	-14.65	-11.22	-9.37	-12.30	1.19
L37A+I15V	-9.09	-11.64	-12.01	-8.75	1.68
L37A+I72V	-11.64	-13.65	-12.99	-10.50	0.60
L37A+Y85A	-8.29	-11.30	-8.46	-9.92	0.40
L37A+I92V	-7.41	-15.49	-14.61	-11.32	1.11
L37A+Y113A	-6.11	-9.84	-7.47	-5.84	1.19
L37A+A130G	-10.59	-12.90	-12.33	-10.50	1.51
G79S+I15V	-12.98	-9.09	-5.05	-9.93	2.40
G79S+I72V	-15.11	-10.59	-6.56	-11.75	1.60
G79S+Y85A	-11.26	-8.25	-3.00	-4.21	1.85
G79S+I92V	-10.68	-12.23	-8.96	-9.11	1.68
G79S+Y113A	-9.29	-7.29	-1.27	-2.95	1.76
G79S+A130G	-15.41	-9.92	-7.72	-9.92	2.34

Table S4: Experimental(28) and calculated staphyloccocal nuclease unfolding free energy nonadditivities (δ_{WT}^{AB}). All values are provided in kJ/mol. Experimental uncertainty 1.26 kJ/mol.

Mut	exp	FoldX	MAESTROweb	Alchemy	err(Alchemy)
I15V+I72V	2.38	0.21	0.07	0.40	0.18
I15V+Y85A	0.67	0.00	-0.71	-0.25	0.51
I15V+Y113A	0.66	0.00	-1.97	0.23	0.50
I72V+Y85A	2.43	0.00	-0.76	1.90	1.60
I72V+Y113A	3.34	0.00	-0.68	3.34	1.70
Y85A+Y113A	0.63	1.17	-1.35	-0.65	0.35
I18M+T33S	-2.10	-0.67	-1.77	-0.75	1.56
I18M+A69T	-0.72	-0.13	-0.95	-1.69	0.76
I18M+A90S	-1.26	6.49	-1.72	-3.35	0.90
T33S+A90S	-1.10	6.36	0.08	-0.06	1.25
A69T+A90S	-0.72	6.15	0.80	-2.18	1.49
V23F+T33S	2.42	6.36	0.68	1.57	0.13
V23F+A69T	3.89	6.24	1.91	5.13	0.81
V23F+A90S	3.72	7.08	1.13	-0.43	1.11
L37A+T33S	1.96	0.00	2.09	1.53	0.32
L37A+A69T	0.75	0.08	3.26	-0.23	0.47
L37A+A90S	1.08	6.11	3.30	1.63	1.51
L7A+V23F	0.97	0.25	-0.03	-0.17	1.94
L7A+L37A	6.74	0.08	2.88	3.25	0.87
L7A+G79S	6.16	3.31	0.55	5.14	2.09
L37A+P117L	-0.88	-0.25	6.25	-1.50	0.55
T62A+G88V	0.42	-3.10	8.70	0.47	2.21
V66L+G88V	-4.19	-3.10	2.31	0.49	0.81
G79S+P117L	1.59	-0.92	2.09	2.75	3.95
V23F+L37A	3.39	5.57	4.18	4.29	1.02
V23F+G79S	0.88	5.61	-1.57	4.05	0.94
L37A+G79S	10.51	-0.80	2.78	6.85	1.44
T62A+V66L	0.42	0.71	2.96	0.69	0.32
L7A+I15V	1.30	0.00	-1.04	2.19	0.92
L7A+I72V	3.94	0.00	1.12	2.45	1.30
L7A+Y85A	1.64	0.00	-1.09	1.46	1.55
L7A+I92V	2.26	-0.04	2.16	0.95	0.21
L7A+Y113A	1.88	1.13	-0.77	0.67	0.82
L7A+A130G	0.97	-0.04	0.05	0.85	0.69
V23F+I15V	2.18	5.19	2.06	2.05	0.81
V23F+I72V	4.90	5.78	1.32	3.59	0.76
V23F+Y85A	0.84	5.90	0.21	0.43	1.12
V23F+I92V	3.30	5.95	1.60	6.40	1.37
V23F+Y113A	1.59	-0.04	-0.85	1.46	0.46
V23F+A130G	-0.38	-0.13	3.12	1.38	0.97
L37A+I15V	1.33	-0.04	0.66	4.26	1.49
L37A+I72V	2.80	-0.04	2.04	4.98	0.52
L37A+Y85A	0.46	-0.04	2.56	-1.39	0.11
L37A+I92V	1.71	-0.29	2.32	2.46	0.79
L37A+Y113A	0.79	0.88	2.40	1.45	0.66
L37A+A130G	1.08	-0.04	4.82	2.67	1.49
G79S+I15V	1.55	-0.50	-0.35	3.48	2.35
G79S+I72V	3.43	0.00	0.50	4.71	1.40
G79S+Y85A	1.59	0.00	0.05	4.89	1.82
G79S+I92V	2.55	-0.04	0.00	5.70	1.66
G79S+Y113A	1.71	0.42	0.62	4.02	1.69
G79S+A130G	0.38	-0.08	1.45	3.93	2.33

Table S5: Experimental (29) and calculated myoglobin folding free energy differences for single mutations ($\Delta\Delta G^{\mathrm{A}}_{\mathrm{WT}}$). All values are provided in kJ/mol.

Mut	exp	FoldX	MAESTROweb	Alchemy	err(Alchemy)
L11A	1.84	13.15	2.37	8.38	0.26
V13A	2.8	10.88	3.75	3.12	0.13
G23A	4.69	-0.13	-0.68	2.1	0.09
V66A	-3.14	3.16	-0.44	-2.65	0.11
V114A	6.07	8.71	1.28	4.59	0.14
G129A	-4.56	-3.21	-3.46	-6.77	0.07
L137A	7.45	4.36	2.36	1.36	0.19
L149A	6.69	8.33	-0.15	7.49	0.21

Table S6: Experimental (30) and calculated myoglobin folding free energy differences for double mutations ($\Delta\Delta G^{\mathrm{AB}}_{\mathrm{WT}}$). All values are provided in kJ/mol.

Mut	exp	FoldX	MAESTROweb	Alchemy	err(Alchemy)
V66A+G23A	-1.88	3.43	-0.67	0.15	0.17
V66A+V114A	2.59	11.73	1.27	1.42	0.21
G129A+V66A	-5.69	-0.05	-2.68	-9.66	0.14
V66A+L149A	4.06	11.42	-0.37	4.16	0.29
V66A+V13A	-3.6	14.05	4.0	1.3	0.19
L11A+V66A	2.38	16.23	2.29	6.74	0.35
V66A+L137A	1.76	7.52	1.57	-1.27	0.25
G129A+V114A	-3.85	5.5	-1.97	-2.76	0.17

Table S7: Experimental (29, 30) and calculated myoglobin folding free energy nonadditivities ($\delta^{\mathrm{AB}}_{\mathrm{WT}}$). All values are provided in kJ/mol.

Mut	exp	FoldX	MAESTROweb	Alchemy Amber99sb*ILDN	err(Alchemy) Amber99sb*ILDN	Alchemy Charmm36m	err(Alchemy) Charmm36m
V66A+G23A	-3.43	0.4	0.44	0.16	0.27	1.43	0.02
V66A+V114A	-0.33	-0.14	0.43	-0.47	0.27	1.41	0.13
G129A+V66A	2.01	0.0	1.21	-0.41	0.31	-0.18	0.15
V66A+L149A	0.5	-0.08	0.21	-0.57	0.15	1.85	0.36
V66A+V13A	-3.26	0.01	0.69	0.65	0.6	0.55	0.4
L11A+V66A	3.68	-0.08	0.36	0.32	0.45	0.19	0.69
V66A+L137A	-2.55	-0.0	-0.35	-0.11	0.42	0.47	0.22
G129A+V114A	-5.36	0.0	0.21	-0.58	0.35	0.2	0.24

Table S8: Experimental and calculated barnase folding free energy differences for single mutations ($\Delta\Delta G^{A}_{WT}$). The experimental values collected from the publications: (31, 33–37). All values are provided in kJ/mol.

Mut	exp	FoldX	MAESTROweb	Alchemy	err(Alchemy)
Y13A	15.52, 12.55, 15.44, 15.52	8.89	9.06	15.89	0.3
Y17A	10.17, 6.99, 9.25, 9.46	5.4	3.42	7.88	0.21
Y13F	2.64	-2.28	1.52	3.45	0.06
Y17F	2.43	-1.26	1.16	0.45	0.06
T16S	7.03, 7.11, 5.69, 7.82	3.5	0.0	-3.35	0.16
T16A	2.09, 1.42	4.37	-1.54	6.83	0.12
H18A	8.45, 8.03	8.21	4.1	0.37	0.2
W94L	6.99	1.4	2.59	2.8	0.25
H18S	8.87	9.17	1.59	-4.05	0.18
H18N	7.61, 6.78	6.02	0.6	5.23	0.14
H18G	4.14, 3.01	3.55	2.32	-0.13	0.22
I4A	6.28, 3.14	9.21	5.94	0.99	0.15
I76V	3.68, 4.1	3.48	5.63	3.15	0.14
Y78F	4.77, 5.9	0.68	4.64	7.35	0.07
I51V	4.73, 7.74	5.03	5.65	11.09	0.11

Table S9: Experimental and calculated barnase folding free energy differences for double mutations ($\Delta\Delta G^{AB}_{WT}$). The experimental values collected from the publications: (31, 33–38). All values are provided in kJ/mol.

Mut	exp	FoldX	MAESTROweb	Alchemy	err(Alchemy)
Y13A+Y17A	20.92, 16.57, 19.92, 19.41	11.36	6.18	14.61	0.58
Y13F+Y17F	5.15	-3.67	1.33	2.05	0.09
T16S+Y17A	10.67	7.74	1.01	4.9	0.3
T16A+Y17A	10.96, 8.03	9.68	0.65	13.21	0.25
H18A+W94L	12.01, 9.71	8.93	3.46	-2.95	0.36
H18S+W94L	11.8	8.4	4.26	-4.92	0.32
H18N+W94L	8.58, 7.2	5.88	4.72	6.84	0.34
H18G+W94L	5.15	3.62	4.41	-2.62	0.37
I4A+I76V	7.78	12.92	8.12	4.03	0.27
I4A+Y78F	10.59	9.89	7.76	6.47	0.15
I4A+I51V	11.0	14.26	9.36	11.97	0.22

Table S10: Experimental and calculated barnase folding free energy nonadditivities (δ^{AB}_{WT}). The nonadditivities were calculated from all combinations of the free energy changes upon single and double mutations in the Tables S8 and S9, respectively. All values are provided in kJ/mol.

Mut	exp	FoldX	MAESTROweb	Alchemy	err(Alchemy)
Y13A+Y17A	-4.77, -1.59, -3.85, -4.06, -1.8, 1.38, -0.88, -1.09, -4.69, -1.51, -3.77, -3.97, -4.77, -1.59, -3.85, -4.06, -9.12, -5.94, -8.2, -8.41, -6.15, -2.97, -5.23, -5.44, -9.04, -5.86, -8.12, -8.33, -9.12, -5.94, -8.2, -8.41, -5.77, -2.59, -4.85, -5.06, -2.8, 0.38, -1.88, -2.09, -5.69, -2.51, -4.77, -4.98, -5.77, -2.59, -4.85, -5.06, -6.28, -3.1, -5.36, -5.56, -3.31, -0.13, -2.38, -2.59, -6.19, -3.01, -5.27, -5.48, -6.28, -3.1, -5.36, -5.56	-2.93	-6.3	-7.54	0.81
Y13F+Y17F	0.08	-0.12	-1.35	-1.28	0.46
T16S+Y17A	-6.53, -3.35, -5.61, -5.82, -6.61, -3.43, -5.69, -5.9, -5.19, -2.01, -4.27, -4.48, -7.32, -4.14, -6.4, -6.61	-1.16	-2.41	0.35	1.09
T16A+Y17A	-1.3, 1.88, -0.38, -0.59, -0.63, 2.55, 0.29, 0.08, -4.23, -1.05, -3.31, -3.51, -3.56, -0.38, -2.64, -2.85	-0.09	-1.23	-1.62	0.13
H18A+W94L	-3.43, -3.01, -5.73, -5.31	-0.68	-3.23	-6.03	0.2
H18S+W94L	-4.06	-2.17	0.08	-3.51	0.09
H18N+W94L	-6.02, -5.19, -7.41, -6.57	-1.54	1.53	-2.08	0.87
H18G+W94L	-5.98, -4.85	-1.33	-0.5	-6.22	0.72
I4A+I76V	-2.18, -2.59, 0.96, 0.54	0.23	-3.45	0.52	0.55
I4A+Y78F	-0.46, -1.59, 2.68, 1.55	0.0	-2.82	-1.0	0.45
I4A+I51V	0.0, -3.01, 3.14, 0.13	0.01	-2.23	-0.12	0.64

References

1. D. Van Der Spoel, E. Lindahl, B. Hess, G. Groenhof, A. E. Mark, and H. J. C. Berendsen. Gromacs: Fast, flexible, and free. *J. Comput. Chem.*, 26(16):1701–1718, 2005.
2. M. J. Abraham, T. Murtola, R. Schulz, S. Páll, J. C. Smith, B. Hess, and E Lindahl. Gromacs: High performance molecular simulations through multi-level parallelism from laptops to supercomputers. *SoftwareX*, 2:1–7, 2015.
3. T. R. Hynes and R. O. Fox. The crystal structure of staphylococcal nuclease refined at 1.7 Å resolution. *Proteins Struct. Funct. Bioinf.*, 10(2):92–105, 1991.
4. G. S. Kachalova, A. N. Popov, and H. D. Bartunik. A steric mechanism for inhibition of CO binding to heme proteins. *Science*, 284(5413):473–476, 1999.
5. C. Martin, V. Richard, M. Salem, R. Hartley, and Y. Mauguen. Refinement and structural analysis of barnase at 1.5 å resolution. *Acta Crystallogr., Sect. D: Biol. Crystallogr.*, 55(2):386–398, 1999.
6. W. L. Jorgensen, J. Chandrasekhar, J. D. Madura, R. W. Impey, and M. L. Klein. Comparison of simple potential functions for simulating liquid water. *J. Chem. Phys.*, 79(2):926–935, 1983.
7. In Suk Joung and Thomas E Cheatham III. Determination of alkali and halide monovalent ion parameters for use in explicitly solvated biomolecular simulations. *J. Phys. Chem. B*, 112(30):9020–9041, 2008.
8. V. Hornak, R. Abel, A. Okur, B. Strockbine, A. Roitberg, and C. Simmerling. Comparison of multiple Amber force fields and development of improved protein backbone parameters. *Proteins Struct. Funct. Bioinf.*, 65(3):712–725, 2006.
9. R. B. Best and G. Hummer. Optimized molecular dynamics force fields applied to the helix-coil transition of polypeptides. *J. Phys. Chem. B*, 113(26):9004–9015, 2009.
10. K. Lindorff-Larsen, S. Piana, K. Palmo, P. Maragakis, J. L. Klepeis, R. O. Dror, and D. E. Shaw. Improved side-chain torsion potentials for the Amber ff99SB protein force field. *Proteins Struct. Funct. Bioinf.*, 78(8):1950–1958, 2010.
11. V. Gapsys, S. Michielssens, D. Seeliger, and B. L. de Groot. Accurate and rigorous prediction of the changes in protein free energies in a large-scale mutation scan. *Angew. Chem. Int. Ed.*, 55:7364–7368, 2016.
12. M. Aldeghi, V. Gapsys, and B. L. de Groot. Accurate estimation of ligand binding affinity changes upon protein mutation. *ACS Cent. Sci.*, 4:1708–1718, 2018.
13. P. Li and K. M. Merz Jr. MCPB.py: A Python Based Metal Center Parameter Builder. *J. Chem. Inf. Model.*, 56(4):599–604, 2016.
14. D.A. Case, D.S. Cerutti, T.E. Cheatham III, T.A. Darden, R.E. Duke, T.J. Giese, H. Gohlke, A.W. Goetz, D. Greene, N. Homeyer, S. Izadi, A. Kovalenko, T.S. Lee, S. LeGrand, P. Li, C. Lin, J. Liu, T. Luchko, R. Luo, D. Mermelstein, K.M. Merz, G. Monard, H. Nguyen, I. Omelyan, A. Onufriev, F. Pan, R. Qi, D.R. Roe, A. Roitberg, C. Sagui, C.L. Simmerling, W.M. Botello-Smith, J. Swails, R.C. Walker, J. Wang, R.M. Wolf, X. Wu, L. Xiao, York D.M., and P.A. Kollman. AMBER 2017, 2017. University of California, San Francisco.
15. M. J. Frisch, G. W. Trucks, H. B. Schlegel, G. E. Scuseria, M. A. Robb, J. R. Cheeseman, G. Scalmani, V. Barone, B. Mennucci, G. A. Petersson, H. Nakatsuji, M. Caricato, X. Li, H. P. Hratchian, A. F. Izmaylov, J. Bloino, G. Zheng, J. L. Sonnenberg, M. Hada, , M. Ehara, K. Toyota, R. Fukuda, Hasegawa, M. Ishida, T. Nakajima, Y. Honda, O. Kitao, H. Nakai, T. Vreven, J. A. Montgomery Jr., J. E. Peralta, F. Ogliaro, M. Bearpark, J. J. Heyd, E. Brothers, K. N. Kudin, V. N. Staroverov, T. Keith, R. Kobayashi, J. Normand, K. Raghavachari, A. Rendell, J. C. Burant, S. S. Iyengar, J. Tomasi, M. Cossi, N. Rega, J. M. Millam, M. Klene, J. E. Knox, J. B. Cross, V. Bakken, C. Adamo, J. Jaramillo, R. Gomperts, R. E. Stratmann, O. Yazyev, A. J. Austin, R. Cammi, C. Pomelli, J. W. Ochterski, R. L. Martin, K. Morokuma, V. G. Zakrzewski, G. A. Voth, P. Salvador, J. J. Dannenberg, S. Dapprich, A. D. Daniels, O. Farkas, J. B. Foresman, J. V. Ortiz, J. Cioslowski, and D. J. Fox. Gaussian 09, Revision C.01. Gaussian Inc. Wallingford CT 2009.

16. J. Huang, S. Rauscher, G. Nawrocki, T. Ran, M. Feig, B. L. De Groot, H. Grubmüller, and A. D. MacKerell. CHARMM36m: an improved force field for folded and intrinsically disordered proteins. *Nat. Methods*, 14(1):71–73, 2017.

17. Giovanni Bussi, Davide Donadio, and Michele Parrinello. Canonical sampling through velocity rescaling. *The Journal of Chemical Physics*, 126(1):014101, January 2007.

18. M. Parrinello and A. Rahman. Polymorphic transitions in single crystals: A new molecular dynamics method. *J. Appl. Phys.*, 52(12):7182–7190, 1981.

19. T. Darden, D. York, and L. Pedersen. Particle mesh Ewald: An Nlog(N) method for Ewald sums in large systems. *J. Chem. Phys.*, 98(12):10089–10092, 1993.

20. U. Essmann, L. Perera, M. L. Berkowitz, T. Darden, H. Lee, and L. G. Pedersen. A smooth particle mesh Ewald method. *J. Chem. Phys.*, 103(19):8577–8593, 1995.

21. B. Hess, H. Bekker, H. J. C. Berendsen, and J. G. E. M. Fraaije. LINCS: a linear constraint solver for molecular simulations. *J. Comput. Chem.*, 18(12):1463–1472, 1997.

22. T. C. Beutler, A. E. Mark, R. C. van Schaik, P. R. Gerber, and W. F. van Gunsteren. Avoiding singularities and numerical instabilities in free energy calculations based on molecular simulations. *Chem. Phys. Lett.*, 222(6):529–539, 1994.

23. V. Gapsys, S. Michielssens, D. Seeliger, and B. L. de Groot. pmx: Automated protein structure and topology generation for alchemical perturbations. *J. Comput. Chem.*, 36:348–354, 2015.

24. M. R. Shirts, E. Bair, G. Hooker, and V. S. Pande. Equilibrium free energies from nonequilibrium measurements using maximum-likelihood methods. *Phys. Rev. Lett.*, 91:140601, 2003.

25. G. E. Crooks. Entropy production fluctuation theorem and the nonequilibrium work relation for free energy differences. *Phys. Rev. E*, 60:2721–2726, 1999.

26. L. Wang, Y. Deng, J. L. Knight, Y. Wu, B. Kim, W. Sherman, J. C. Shelley, T. Lin, and R. Abel. Modeling local structural rearrangements using fep/rest: Application to relative binding affinity predictions of cdk2 inhibitors. *J. Chem. Theory Comput.*, 9(2):1282–1293, 2013.

27. D. Markthaler, G. Kraus, and N. Hansen. Overcoming convergence issues in free-energy calculations of amide-to-ester mutations in the pin1-ww domain. *J. Chem. Inf. Model*, 58(11):2305–2318, 2018.

28. R. J. Pinker, L. Lin, N. R. Kallenbach, and G. D. Rose. Effects of alanine substitutions in α-helices of sperm whale myoglobin on protein stability. *Protein Sci.*, 2(7):1099–1105, 1993.

29. L. Lin, R. J. Pinker, G. N. Phillips, and N. R. Kallenbach. Stabilization of myoglobin by multiple alanine substitutions in helical positions. *Protein Sci.*, 3(9):1430–1435, 1994.

30. S. M. Green and D. Shortle. Patterns of nonadditvity between pairs of stability mutations in staphylococcal nuclease. *Biochemistry*, 32:10131–10139, 1993.

31. L. Serrano, J. T. Kellis Jr., P. Cann, A. Matouschek, and A. R. Fersht. The folding of an enzyme: II. Substructure of barnase and the contribution of different interactions to protein stability. *J. Mol. Biol.*, 224(3):783–804, 1992.

32. C. N. Pace and J. Hermans. The stability of globular protein. *CRC critical reviews in biochemistry*, 3(1):1–43, 1975.

33. R. Loewenthal, J. Sancho, and A. R. Fersht. Histidine-aromatic interactions in barnase: Elevation of histidine pKa and contribution to protein stability. *J. Mol. Biol.*, 224(3):759–770, 1992.

34. L. Serrano, A. Matouschek, and A. R. Fersht. The folding of an enzyme: III. Structure of the transition state for unfolding of barnase analysed by a protein engineering procedure. *J. Mol. Biol.*, 224(3):805–818, 1992.

35. A. Matouschek, L. Serrano, and A. R. Fersht. The folding of an enzyme: IV. Structure of an intermediate in the refolding of barnase analysed by a protein engineering procedure. *J. Mol. Biol.*, 224(3):819–835, 1992.

36. L. Serrano, M. Bycroft, and A. R. Fersht. Aromatic-aromatic interactions and protein stability: investigation by double-mutant cycles. *J. Mol. Biol.*, 218(2):465–475, 1991.

37. L. Serrano, J. Sancho, M. Hirshberg, and A. R. Fersht. α-Helix stability in proteins: I. Empirical correlations concerning substitution of side-chains at the N and C-caps and the replacement of alanine by glycine or serine at solvent-exposed surfaces. *J. Mol. Biol.*, 227(2):544–559, 1992.

38. J. M. Sanz and A. R. Fersht. Rationally designing the accumulation of a folding intermediate of barnase by protein engineering. *Biochemistry*, 32(49):13584–13592, 1993.

39. D. Shortle, W. E. Stites, and A. K. Meeker. Contributions of the large hydrophobic amino acids to the stability of staphylococcal nuclease. *Biochemistry*, 29:8033–8041, 1990.
